# The Regulation of the AdcR Regulon in *Streptococcus pneumoniae* Depends Both on Zn^2+^- and Ni^2+^-Availability

**DOI:** 10.3389/fcimb.2015.00091

**Published:** 2015-12-08

**Authors:** Irfan Manzoor, Sulman Shafeeq, Muhammad Afzal, Oscar P. Kuipers

**Affiliations:** ^1^Department of Molecular Genetics, Groningen Biomolecular Sciences and Biotechnology Institute, University of GroningenGroningen, Netherlands; ^2^Department of Bioinformatics and Biotechnology, Government College University FaisalabadFaisalabad, Pakistan; ^3^Department of Microbiology, Tumor and Cell Biology, Karolinska InstitutetStockholm, Sweden

**Keywords:** metal homeostasis, pneumococcus, nickel, zinc, AdcR, Pht family proteins, AdcR regulon, PsaR regulon

## Abstract

By using a transcriptomic approach, we have elucidated the effect of Ni^2+^ on the global gene expression of *S. pneumoniae* D39 by identifying several differentially expressed genes/operons in the presence of a high extracellular concentration of Ni^2+^. The genes belonging to the AdcR regulon (*adcRCBA, adcAII*-*phtD, phtA, phtB*, and *phtE*) and the PsaR regulon (*pcpA, prtA*, and *psaBCA*) were highly upregulated in the presence of Ni^2+^. We have further studied the role of Ni^2+^ in the regulation of the AdcR regulon by using ICP-MS analysis, electrophoretic mobility shift assays and transcriptional *lacZ*-reporter studies, and demonstrate that Ni^2+^ is directly involved in the derepression of the AdcR regulon *via* the Zn^2+^-dependent repressor AdcR, and has an opposite effect on the expression of the AdcR regulon compared to Zn^2+^.

## Introduction

In bacteria, the transition metal ions play an important role in the proper functioning of many enzymes, transporters, and transcriptional regulators. Transition metal ions are the prerequisite for the proper bacterial growth at low concentrations, but metal ions can be lethal at higher concentrations (Blencowe and Morby, 2003; Finney and O'Halloran, 2003; Moore and Helmann, 2005; Ge et al., 2012). Therefore, proper homeostasis of metal ions is very important for the survival of bacteria, which is maintained by the dedicated metal transport- and efflux-systems (Tottey et al., 2008; Waldron and Robinson, 2009; Lisher et al., 2013). These systems are tightly regulated by metal-responsive transcriptional regulators to ensure the proper functioning of the cell by maintaining the minimum levels of metal ions inside the cell.

*Streptococcus pneumoniae* is one of the most common human pathogens that reside asymptomatically in the human nasopharynx (Mitchell, 2003). However, it may occasionally translocate to the lungs, the eustachian tube, the blood, and the nervous system, causing pneumoniae, otitis media, bacteremia, and meningitis, respectively (Obaro and Adegbola, 2002; Bogaert et al., 2004). During translocation from the nasopharynx to other infection sites, *S. pneumoniae* may encounter different environmental conditions including varying metal ions concentrations, which might affect the expression of different genes including virulence genes (Gupta et al., 2009; Shafeeq et al., 2011b, 2013; Plumptre et al., 2014a). However, the exact conditions that *S. pneumoniae* might face during infections, are poorly understood.

The role of manganese (Mn^2+^), zinc (Zn^2+^), copper (Cu^2+^), iron (Fe^2+^), cobalt (Co^2+^), and cadmium (Cd^2+^) on the gene regulation of *S. pneumoniae* have already been established and several metal-specific acquisition- and efflux-systems have been characterized. These systems include AdcRCBA (the Zn^2+^-uptake system), CzcD (the Zn^2+^-efflux system), PsaBCA (the Mn^2+^-uptake system), MntE (the Mn^2+^-efflux system), the *cop* operon (the Cu^2+^-efflux system), and PiaABCD, PiuBCDA, and PitADBC (the Fe^2+^- and Fe^3+^-uptake systems) (Kloosterman et al., 2007, 2008; Hendriksen et al., 2009; Rosch et al., 2009; Bayle et al., 2011; Shafeeq et al., 2011a, 2013; Manzoor et al., 2015c). These systems have further been shown to be regulated by metal-specific transcriptional regulators in *S. pneumoniae*. The Zn^2+^-uptake system (AdcRCBA) is repressed by transcriptional regulator AdcR in the presence of Zn^2+^ (Shafeeq et al., 2011a). Similarly, the *psaBCA* operon encoding Mn^2+^-uptake system are repressed by transcriptional regulator PsaR in the presence of Mn^2+^ (Johnston et al., 2006; Kloosterman et al., 2008), whereas, this PsaR-mediated repression is relieved by the addition of Zn^2+^, Co^2+^, Cd^2+^, or Ni^2+^ (Kloosterman et al., 2008; Jacobsen et al., 2011; Begg et al., 2015; Manzoor et al., 2015a,b,c).

Ni^2+^ is an essential micronutrient for certain bacteria, due to its role in various cellular processes like methane formation, hydrolysis of urea, and consumption of molecular hydrogen (Chen and Burne, 2003; Mulrooney and Hausinger, 2003; Rodionov et al., 2006; Anwar et al., 2007). In *Escherichia coli*, the *nik* operon (*nikABCDE*) involved in the transport of Ni^2+^ is shown to regulate by transcriptional regulator NikR (De Pina et al., 1999). Moreover, the expression of NmtA, an ATP-dependent transporter involved in the efflux of Ni^2+^ and Co^2+^, is tightly regulated by Ni^2+^-responsive transcriptional regulator NmtR in *Mycobacterium tuberculosis* (Cavet et al., 2002). Ni^2+^ is also shown to regulate the expression of urease activity in *Streptococcus salivarius* and *Helicobacter pylori* (van Vliet et al., 2001; Chen and Burne, 2003). The amount of Ni^2+^ in the human blood is estimated to be 0.83 ng ml^−1^ (Alimonti et al., 2005) and it is likely that *S. pneumoniae* may encounter Ni^2+^ during infection in blood. So far, very little is known about the impact of Ni^2+^ on the global gene expression of *S. pneumoniae*. Previously, the role of Ni^2+^ in the regulation of the Zn^2+^-efflux system *czcD* was reported (Kloosterman et al., 2007). It was shown that the SczA-mediated expression of *czcD* was highly increased in the presence of Zn^2+^, Co^2+^, or Ni^2+^ (Kloosterman et al., 2007). Moreover, a number of proteins and motif with Co^2+^- and Ni^2+^-binding capacity has been identified by Immobilized metal affinity column (IMAC) and LTQ-Orbitrap mass spectrometry (MS) that have diverse functions in the *S. pneumoniae* (Sun et al., 2013). In a recent study, we demonstrated the role of Ni^2+^ in regulation of the PsaR regulon and showed that Ni^2+^ not only alleviates the Mn^2+^-dependent binding of PsaR to the promoter regions of the PsaR regulon genes, but also cause Mn^2+^ deficiency possibly by blocking Mn^2+^-uptake *via* PsaA, hence leading to the high expression of the PsaR regulon in the presence of Ni^2+^ (Manzoor et al., 2015b).

In this current study, we used a transcriptomic analysis approach for the identification of differentially expressed genes/operons in response to high extracellular Ni^2+^ in *S. pneumoniae*. The expression of genes belonging to the AdcR regulon and the PsaR regulon was highly upregulated in the presence of Ni^2+^. We further studied the role of Ni^2+^ in the AdcR-mediated regulation of the *adcRCBA, adcAII*-*phtD, phtA, phtB*, and *phtE* by using transcriptional *lacZ*-reporter studies, inductively coupled plasma-mass spectrometry (ICP-MS) analysis and electrophoretic mobility shift assays (EMSAs), and showed that Ni^2+^ and Zn^2+^ play an opposite role in the regulation of the *adcRCBA, adcAII*-*phtD, phtA, phtB*, and *phtE*.

## Materials and methods

### Bacterial strains and media

Bacterial strains used in this study are listed in Table 1. Growth of bacteria and DNA manipulation were performed as described (Shafeeq et al., 2011a; Manzoor et al., 2015a). All experiments in this study were performed in chemically defined medium (CDM). Salts of metal ion ZnSO_4_.7H_2_O and NiSO_4_.6H_2_O were used as specified in the Results section. Primers used in this study are based on the genome sequence of *S. pneumoniae* D39 and are listed in Table 2.

**Table 1:** **List of strains and plasmids used in this study**.

**Strain/plasmid**	**Description**	**Source**
***S. pneumoniae***		
D39	Serotype 2 strain, *cps 2*	Laboratory of P. Hermans
SS200	D39 Δ*adcR*; Ery^R^	Shafeeq et al., 2011a
IM404	D39 Δ*bgaA*::P*czcD-lacZ*; Tet^R^	Manzoor et al., 2015a
IM501	D39 Δ*bgaA*::P*adcR-lacZ*; Tet^R^	This study
IM502	D39 Δ*bgaA*::P*adcAII-lacZ*; Tet^R^	This study
IM503	D39 Δ*bgaA*::P*phtA-lacZ*; Tet^R^	This study
IM504	D39 Δ*bgaA*::P*phtB-lacZ*; Tet^R^	This study
IM505	D39 Δ*bgaA*::P*phtE-lacZ*; Tet^R^	This study
IM551	SS200 Δ*bgaA*::P*adcR-lacZ*; Tet^R^	This study
IM552	SS200 Δ*bgaA*::P*adcAII-lacZ*; Tet^R^	This study
IM553	SS200 Δ*bgaA*::P*phtA-lacZ*; Tet^R^	This study
IM554	SS200 Δ*bgaA*::P*phtB-lacZ*; Tet^R^	This study
IM555	SS200 Δ*bgaA*::P*phtE-lacZ*; Tet^R^	This study
***E. coli***		
EC1000	Km^R^; MC1000 derivative carrying a single copy of the pWV1 *repA* gene in *glgB*	Laboratory collection
***Plasmids***		
pPP2	Amp^R^ Tet^R^; promoterless *lacZ* For replacement of *bgaA* with promoter *lacZ*-fusion. Derivative of pPP1	Halfmann et al., 2007
pIM501	pPP2 P*adcR-lacZ*	This study
pIM502	pPP2 P*adcAII-lacZ*	This study
pIM503	pPP2 P*phtA-lacZ*	This study
pIM504	pPP2 P*phtB-lacZ*	This study
pIM505	pPP2 P*phtE-lacZ*	This study
SS107	pNZ8048 carrying strep-tagged AdcR downstream of PnisA	Shafeeq et al., 2011a

**Table 2:** **List of primers used in this study**.

**Name**	**Nucleotide sequence (5′ 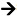 3′)**	**Restriction site**
Padcr-F	CGGAATTCTTTTTCAGCAAAGATTGGG	EcoRI
Padcr-R	CGGGATCCCTTTCCTTTTAGACTTCTC	BamHI
PadcAII-F	CGGAATTCCTTCACTTATGGCTATAAGC	EcoRI
PadcAII-R	CGGGATCCAAAGAAAGACACTTAACAGG	BamHI
PphtA-F	CGGAATTCTGAACTTCAAAAAGAATACG	EcoRI
PphtA-R	CGGGATCCCTTAAAATCAAAGCTGCCGC	BamHI
PphtB-F	GCATGAATTCGGCAGAAGCAGAAAAATTAC	EcoRI
PphtB-R	CGATGGATCCAAGTGTAGCTACTGACC	BamHI
PphtE-F	CGGAATTCAGAAGTAGATAGTCTCTTGG	EcoRI
PphtE-R	CGGGATCCACGATAACAGCTGATCCAGC	BamHI

### DNA microarray and data analysis

For microarray analysis in response to Ni^2+^, *S. pneumoniae* D39 wild-type was grown in two biological replicates in CDM with and without the addition of 0.5 mM NiSO_4._6H_2_O. To analyze the impact of *adcR* deletion on the transcirptome of *S. pneumoniae* in the presence of Ni^2+^, D39 wild-type and Δ*adcR* (SS200) (Shafeeq et al., 2011a) were grown in two biological replicates in CDM with 0.3 mM of NiSO_4._6H_2_O. All other procedures regarding microarray experiments and data analysis were done as described before (Shafeeq et al., 2011b; Afzal et al., 2015). For the identification of differentially expressed genes a Bayesian *p* < 0.001 and a fold change cut-off of 2 was applied. The DNA microarray data have been submitted to gene expression omnibus (GEO) database under the accession number GSE73852.

### Construction of transcriptional *lacZ*-fusions and β-galactosidase assays

Chromosomal transcriptional *lacZ*-fusions to the promoter regions of *adcR, adcAII, phtA, phtB*, and *phtE* were constructed in plasmid pPP2 (Halfmann et al., 2007) with the primer pairs listed in Table 2, resulting in pIM501-505. These plasmids were introduced into D39 wild-type and Δ*adcR* (SS200) (Shafeeq et al., 2011a) resulting in strains IM501-505 and IM551-554, respectively. All plasmids were checked for the presence of correct insert by means of PCR and DNA sequencing. For β-galactosidase activity, the derivatives of *S. pneumoniae* were grown in triplicate in CDM supplemented with different metal ion concentrations (w/v) mentioned in the Results and harvested at the mid-exponential growth phase. The β-galactosidase activity was measured as described before (Kloosterman et al., 2006). Standard deviations were calculated from three independent replicates of each sample.

### Inductively coupled plasma-mass spectrometry (ICP-MS) analysis

To determine the cell-associated concentration of metal ions, an ICP-MS analysis was performed on the cells grown in triplicates in CDM with and without the addition of 0.5 mM Ni^2+^ till the mid-exponential growth phase. Cell cultures were centrifuged at 4°C and washed twice with overnight Chelex (Sigma) treated phosphate-buffered saline (PBS) with 1 mM nitrilotriacetic acid. Cells were dried overnight in a Speedvac at room temperature. The dried cells were dissolved in 2.5% nitric acid (Ultrapure, Sigma Aldrich) and lysed at 95^*o*^C for 10 min by vigorous vortexing after each 30 s. The lysed cell samples were used for ICP-MS analysis as described (Jacobsen et al., 2011). Metal ion concentrations were expressed as μg g^−1^ dry weight of cells.

### Overexpression and purification of strep-tagged AdcR

The nisin-inducible (NICE) expression system (Kuipers et al., 1998) in *Lactococcus lactis* strain NZ9000 was used for the overexpression of C-terminally Strep-tagged AdcR (Shafeeq et al., 2011a). Cells were grown until an OD_600_ of 0.4 in 1 L culture followed by the induction with 10 ng ml^−1^ nisin. The purification of AdcR-Strep tag was performed using the Streptactin column from IBA according to the supplier's instructions (www.iba-go.com). The purified protein was eluted in buffers without EDTA and stored at a concentration of 0.5 mg/ml in the elution buffer (100 mM Tris-HCl [pH 8], 150 mM NaCl, 2.5 mM desthiobiotin, and 1 mM β-mercaptoethanol) with 10% glycerol at −80°C.

### Electrophoretic mobility shift assays

Electrophoretic mobility shift assays (EMSAs) were performed as described (Kloosterman et al., 2008). In short, PCR products of the promoter regions of *adcR, adcAII, phtA, phtB*, and *pcpA* were labeled with [γ-^33^P] ATP. All the EMSAs were performed with 5000 cpm of [γ-^33^P] ATP-labeled PCR products in buffer containing 20 mM Tris-HCL (pH 8.0), 5 mM MgCl_2_, 8.7% (w/v) glycerol, 62.5 mM KCl, 25 μg/ml bovine serum albumin and 25 μg/ml poly (dI-dC). Various metal ions were added in concentrations as described in the Results section. Reactions were incubated at 30°C for 30 min before loading on gels. Gels were run in 1 M Tris-borate buffer (pH 8.3) at 95 V for 90 min.

## Results

### Identification of Ni^2+^-dependent genes in *S. pneumoniae*

To investigate the impact of Ni^2+^ on the transcriptome of *S. pneumoniae*, a DNA microarray-based comparison of D39 wild-type grown in CDM with 0.5 mM Ni^2+^ to the same strain grown in CDM with 0 mM Ni^2+^ was performed. Table 3 summarizes the list of differentially expressed genes in the presence of 0.5 mM Ni^2+^. The PsaR regulon consisting of the operon *psaBCA* (encoding Mn^2+^-dependent ABC transporters, PsaBCA), *pcpA* (encoding a choline binding protein, PcpA), and *prtA* (encoding a serine protease PrtA) were highly upregulated in the presence of Ni^2+^. The Ni^2+^-dependent upregulation of the PsaR regulon in the presence of Ni^2+^ is consistent with our recent study, where we have explored the Ni^2+^-dependent regulation of the PsaR regulon in more details (Manzoor et al., 2015b). Expression of a gene cluster including the cation efflux system gene *czcD*, the MerR family transcriptional regulator, and the Zn^2+^-containing alcohol dehydrogenase *adhB* was increased more than 35-fold in the presence of Ni^2+^. The cation efflux system CzcD was shown to protect *S. pneumoniae* against the intracellular Zn^2+^-stress (Kloosterman et al., 2007). A novel TetR family transcriptional regulator SczA has been shown to activate the expression of *czcD* in the presence of Zn^2+^, Co^2+^, or Ni^2+^ (Kloosterman et al., 2007). Therefore, the upregulation of *czcD* in our transcriptomic analysis is consistent with the finding presented in previous study (Kloosterman et al., 2007). Furthermore, genes encoding a heat shock protein (HtpX) and a Dpr homolog (spd_1402) were also differentially expressed. The Dpr protein has been shown to protect bacterial cells from oxidative stress (Pulliainen et al., 2003).

**Table 3:** **Summary of transcriptome comparison of ***S. pneumoniae*** D39 wild-type grown in CDM plus 0.5 mM Ni^2+^ to CDM plus 0 mM Ni^2+^**.

**Gene tag^a^**	**Function^b^**	**Ratio^c^**	***P*-value**
*SPD0475*	CAAX amino terminal protease family protein	5.39	1.48E−11
*SPD0526*	Fructose-1,6-bisphosphate aldolase, class II	3.07	1.93E−13
*SPD0558*	Cell wall-associated serine protease, PrtA	9.67	2.92E−13
*SPD0738*	Cytidine deaminase	21.92	6.05E−07
*SPD0888*	Adhesion lipoprotein, AdcAII (LmB)	2.07	7.08E−07
*SPD0889*	Pneumococcal histidine triad protein D, PhtD	2.06	5.81E−10
*SPD0890*	Pneumococcal histidine triad protein E, PhtE	7.13	2.81E−05
*SPD1038*	Pneumococcal histidine triad protein A, PhtA	12.54	2.00E−14
*SPD1078*	L-lactate dehydrogenase	4.21	2.04E−14
*SPD1138*	Heat shock protein, HtpX	3.61	9.44E−05
*SPD1360*	Hypothetical protein	7.29	1.15E−10
*SPD1402*	Non-heme iron-containing ferritin, DpR	2.65	2.10E−11
*SPD1461*	Manganese ABC transporter, ATP-binding protein, PsaB	11.90	5.33E−15
*SPD1462*	Manganese ABC transporter, permease protein, PsaC	10.71	5.26E−14
*SPD1464*	Thiol peroxidase	2.13	8.68E−10
*SPD1632*	Hypothetical protein	2.32	3.47E−05
*SPD1633*	Galactose-1-phosphate uridylyl transferase, GalT	2.68	7.81E−06
*SPD1634*	Galactokinase, GalK	4.13	1.29E−08
*SPD1635*	Galactose operon repressor, GalR	5.00	4.27E−07
*SPD1636*	Alcohol dehydrogenase, zinc-containing, AdhB	35.69	0.00E+00
*SPD1637*	Transcriptional regulator, MerR family	38.25	0.00E+00
*SPD1638*	Cation efflux system protein, CzcD	77.89	5.55E−15
*SPD1651*	Iron-compound ABC transporter, ATP-binding protein	−3.91	1.27E−13
*SPD1652*	Iron-compound ABC transporter, iron-compound-binding protein	−3.73	4.57E−12
*SPD1965*	Choline binding protein, PcpA	2.80	8.16E−04
*SPD1997*	Zinc ABC transporter, zinc-binding lipoprotein, AdcA	3.91	1.14E−12
*SPD1998*	Zinc ABC transporter, permease protein, AdcB	2.00	2.47E−04
*SPD1999*	Zinc ABC transporter, ATP-binding protein, AdcC	4.17	1.29E−13
*SPD2000*	*adc* operon repressor, AdcR	3.88	2.46E−11

a *Gene numbers refer to D39 locus tags*.

b *D39 annotation/TIGR4 annotation (Hoskins et al., 2001; Lanie et al., 2007)*.

c *Ratios >2.0 or < 2.0 (wild-type + 0.5 mM Ni^2+^/wild-type + 0 mM Ni^2+^)*.

The genes belonging to the AdcR regulon were also upregulated in the presence of Ni^2+^. The expression of the *adc* operon was 4-fold upregulated. The expression of *adcAII-phtD* operon was upregulated 2-fold. The expression of other genes encoding for Pht family proteins (PhtA and PhtE), was upregulated more then 7-fold. Previously, it was shown that the expression of the AdcR regulon is repressed by the transcriptional regulator AdcR in the presence of Zn^2+^ (Shafeeq et al., 2011a). Transcriptome data was further validated by qRT-PCR analysis (Supplementary data: Table S1). Upregulation of the AdcR regulon in the presence of Ni^2+^ might also indicate the putative role of Ni^2+^ in the regulation of the AdcR regulon by the transcriptional regulator AdcR. Therefore, we decided to further explore the role of Ni^2+^ in the regulation of the AdcR regulon and to determine the intracellular concentrations of metal ions in *S. pneumoniae* D39 grown in the presence of either 0.5 mM Ni^2+^ or 0 mM Ni^2+^ in CDM.

### *S. pneumoniae* accumulates more Ni^2+^ when grown in the presence of 0.5 mM Ni^2+^

**Figure 1:**
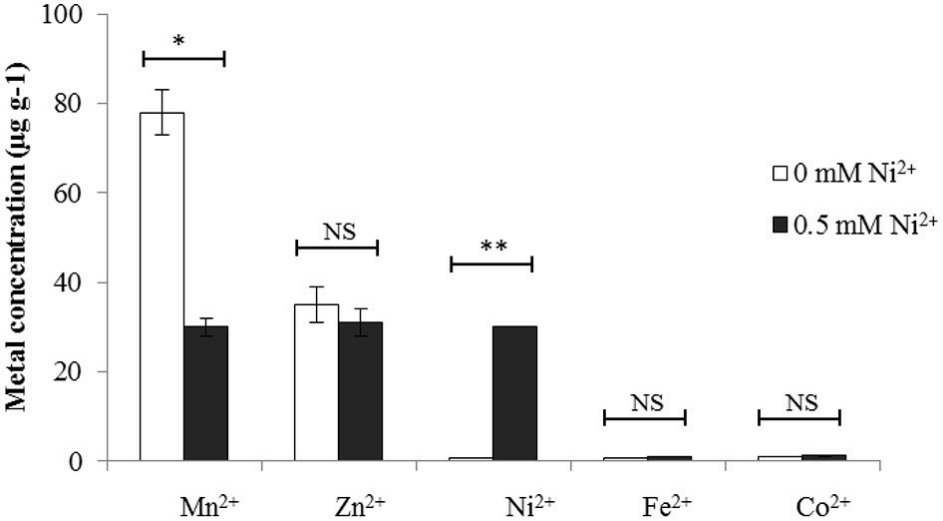
**Cell-associated metal ion concentrations (expressed ug g^−1^) of ***S. pneumoniae*** D39 wild-type when grown in CDM with either 0 mM or 0.5 mM Ni^2+^**. The statistical significance of the differences in the mean metal ion concentrations was determined by One-way ANOVA (NS not significant, ^*^P < 0.05, and ^**^P < 0.001).

To investigate whether the observed transcriptomic responses correlated with high cell-associated concentration of Ni^2+^, we performed an ICP-MS analysis on the same conditions used for performing the transcriptome analysis, i.e., cells grown either in the presence of 0.5 mM Ni^2+^ or 0 mM Ni^2+^ in CDM. Our ICP-MS data revealed that the cells grown in the presence of 0.5 mM Ni^2+^ accumulate 30-fold more cell-associated Ni^2+^ compared to the cells grown in 0 mM Ni^2+^ (30 μg g^−*l*^ dry mass of cells vs. < 1 μg g^−*l*^ dry mass of cells) (Figure 1). Moreover, 2.6-fold decrease in the cell-associated concentration of Mn^2+^ was observed. The cell-associated concentration of other metal ions was not changed in the presence of 0.5 mM Ni^2+^ compared to 0 mM Ni^2+^. Therefore, it is likely that the transcriptomic changes observed in the presence of 0.5 M Ni^2+^ are due to the high intracellular concentration of Ni^2+^.

### Ni^2+^-dependent expression of the AdcR regulon

**Figure 2:**
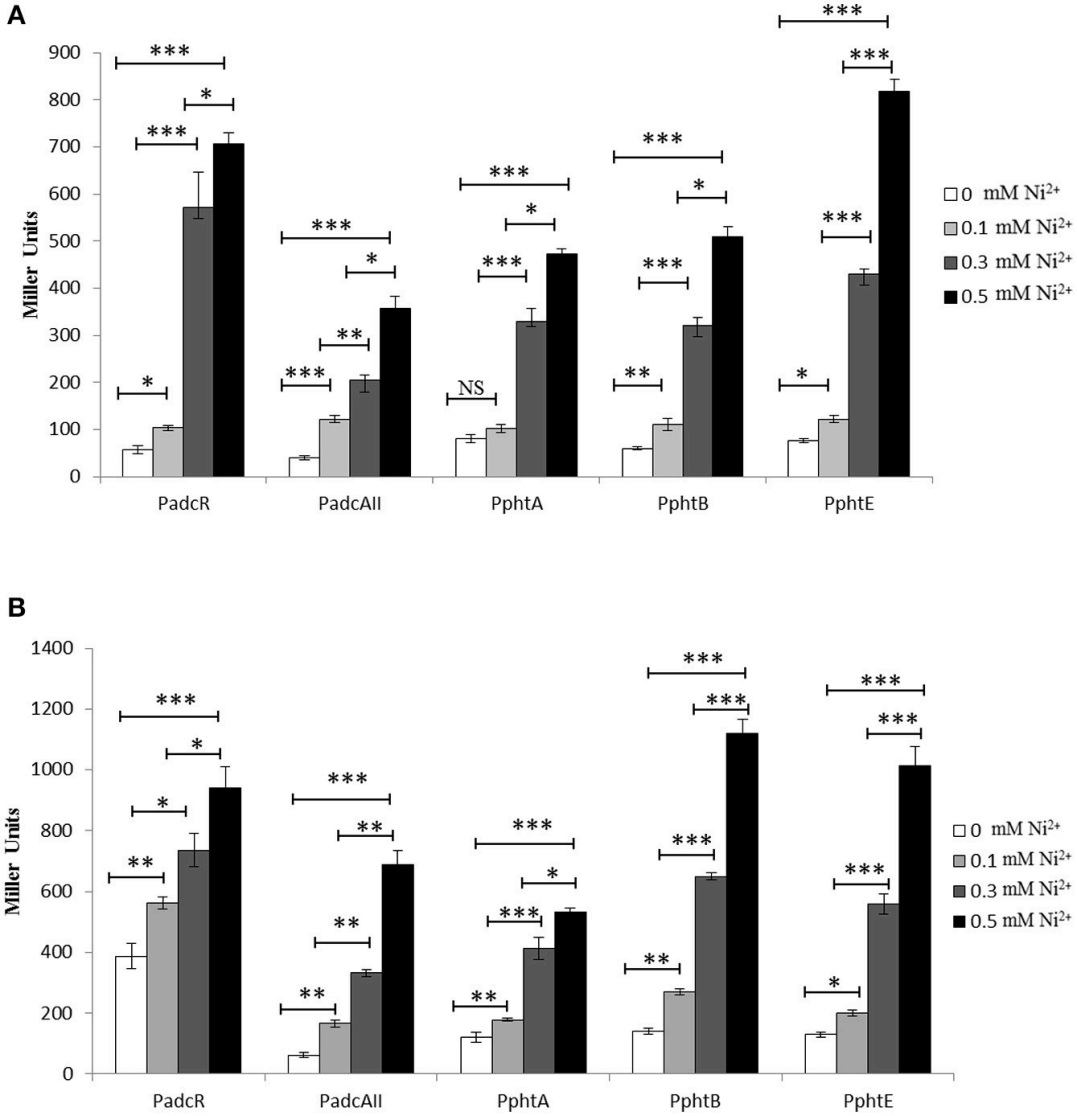
**Expression level (in Miller units) of the D39 wild-type containing transcriptional ***lacZ***-fusions to P***adcR***, P***adcAII***, P***phtA***, P***phtB***, and P***phtE***, grown in CDM (A) and CDM-Zn^2+^ (Zn^2+^-depleted medium) (B) with different added concentrations of Ni^2+^**. Standard deviation of three independent replications is indicated with error bars. Statistical significance of the differences in the expression levels was determined by One-way ANOVA (NS, not significant, ^*^P < 0.05, ^**^P < 0.001, and ^***^P < 0.0001).

To explore the transcriptional regulation of the genes/operons belonging to the AdcR regulon (*adcRCBA, adcAII*-*phtD, phtA, phtB*, and *phtE*) found in our microarray analysis, transcriptional *lacZ*-fusions were constructed to the promoter regions of *adcR, adcAII, phtA, phtB*, and *phtE* in plasmid pPP2 (Halfmann et al., 2007) and transferred to *S. pneumoniae* D39 wild-type. The expression of P*adcR*-*lacZ*, P*adcAII*-*lacZ*, P*phtA*-*lacZ*, P*phtB*-*lacZ*, and P*phtE*-*lacZ* was measured in CDM and CDM-Zn^2+^ (Zn^2+^ depleted medium) with the addition of 0, 0.1, 0.3, or 0.5 mM Ni^2+^. As AdcR represses the expression of the AdcR regulon in the presence of Zn^2+^, we also used Zn^2+^-depleted medium (CDM-Zn^2+^). β-galactosidase activity (Miller Units) showed that the elevated concentration of Ni^2+^ led to the high expression of all these promoters in CDM and CDM-Zn^2+^ (Figures 2A,B). However, the expression of these promoters was much higher in CDM-Zn^2+^ compared to CDM. The full CDM contains minor amounts of Zn^2+^ (around 883 μg l^−1^) (Manzoor et al., 2015a), which could explain the lower expression of these promoters in CDM compared to CDM-Zn^2+^. This data not only suggests the role of Ni^2+^ in the regulation of the *adcRCBA, adcAII*-*phtD, phtA, phtB*, and *phtE*, but also indicate the ability of Ni^2+^ to derepress the Zn^2+^-dependent repression of these genes.

### Opposite effect of Zn^2+^ and Ni^2+^ on the expression of the AdcR regulon

**Figure 3:**
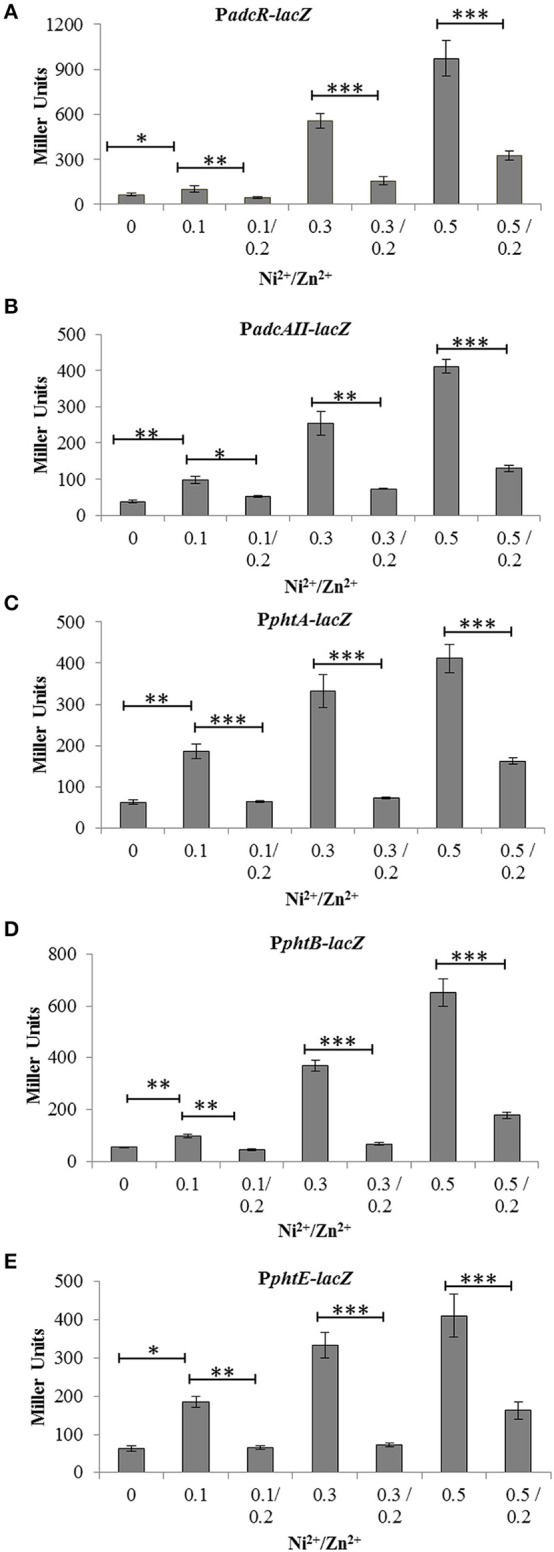
**Expression level (in Miller units) of the D39 wild-type containing transcriptional ***lacZ***-fusions to P***adcR*** (A), P***adcAII*** (B), P***phtA*** (C), P***phtB*** (D), and P***phtE*** (E), grown in CDM with or without addition of different concentrations of Ni^2+^ and Zn^2+^**. Standard deviation of three independent replications is indicated with error bars. Statistical significance of the differences in the expression levels was determined by One-way ANOVA (^*^P < 0.05, ^**^P < 0.001, and ^***^P < 0.0001).

β-galactosidase activities shown above indicate that Ni^2+^ might compete with Zn^2+^ and that both metal ions have opposite effects on the expression of the *adcRCBA, adcAII*-*phtD, phtA, phtB*, and *phtE*. In order to study the interplay of Ni^2+^ and Zn^2+^ in the regulation of *adcRCBA, adcAII*-*phtD, phtA, phtB*, and *phtE* in more details, we performed β-galactosidase assays with P*adcR*-*lacZ*, P*adcAII*-*lacZ*, P*phtA*-*lacZ*, P*phtB*-*lacZ*, and P*phtE*-*lacZ* in CDM with the addition of varying concentrations of Ni^2+^ and Zn^2+^ together. β-galactosidase data (Miller Units) showed that addition of Zn^2+^ in the medium leads to the repression of P*adcR*-*lacZ*, P*adcAII*-*lacZ*, P*phtA*-*lacZ*, P*phtB*-*lacZ*, and P*phtE-lacZ*, even in the presence of Ni^2+^. However, repression caused by Zn^2+^ was much weaker at higher concentrations of Ni^2+^ (Figures 3A–E). This data confirm that Ni^2+^ and Zn^2+^ have an opposite effects on the expression of *adcRCBA, adcAII*-*phtD, phtA, phtB*, and *phtE*, where Zn^2+^ represses and Ni^2+^ derepresses the expression of these genes.

### Role of the transcriptional regulator AdcR in the Ni^2+^-dependent expression of the AdcR regulon

**Figure 4:**
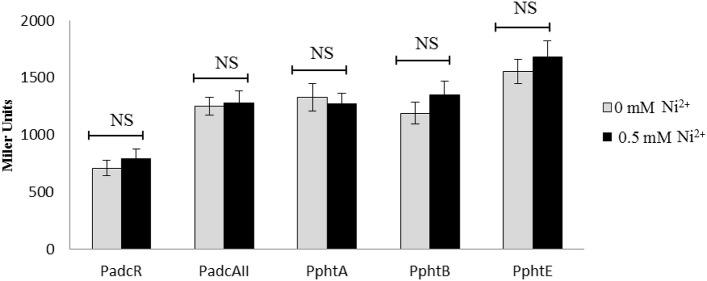
**Expression level (in Miller units) of the ***adcR*** mutant containing transcriptional ***lacZ***-fusions to P***adcR***, P***adcAII***, P***phtA***, P***phtB***, and P***phtE*** grown in CDM with or without addition of 0.5 mM Ni^2+^**. Standard deviation of three independent replications is indicated with error bars. Statistical significance of the differences in the expression levels was determined by One-way ANOVA (NS, not significant).

Previously, it has been shown that the transcriptional regulator AdcR represses the expression of *adcRCBA, adcAII*-*phtD, phtA, phtB*, and *phtE* in the presence of Zn^2+^ (Shafeeq et al., 2011a). In this study, our transcriptomic analysis and transcriptional *lacZ*-reporter data indicate that Ni^2+^ derepresses the expression of these genes. To identify whether the transcriptional regulator AdcR is also responsible for the Ni^2+^-dependent expression of *adcRCBA, adcAII*-*phtD, phtA, phtB*, and *phtE*, we have transformed P*adcR*-*lacZ*, P*adcAII*-*lacZ*, P*phtA*-*lacZ*, P*phtB*-*lacZ*, and P*phtE*-*lacZ* into the *adcR* mutant (SS200) and performed β-galactosidase assays. β-galactosidase data revealed that the deletion of *adcR* leads to increase expression of P*adcR*-*lacZ*, P*adcAII*-*lacZ*, P*phtA*-*lacZ*, P*phtB*-*lacZ*, and P*phtE*-*lacZ* even in the absence of Ni^2+^ (Figure 4). Upregulation of these transcriptional *lacZ*-fusions in the *adcR* mutant indicates that Ni^2+^-dependent expression of *adcRCBA, adcAII*-*phtD, phtA, phtB*, and *phtE* is mediated by transcriptional regulator AdcR.

To elucidate the Ni^2+^-dependent role of AdcR in more details and find more targets of AdcR in the presence of Ni^2+^, microarray comparison of the *adcR* mutant with D39 wild-type was performed in CDM with 0.3 mM Ni^2+^. As expected, the expression of genes belonging to the AdcR regulon was highly upregulated (Table 4), except for the *adc* operon, which was downregulated in our transcriptome analysis (Table 4). For creating an *adcR* mutant in previous study, an erythromycin-resistance gene cassette was used to replace the *adcR* gene (Shafeeq et al., 2011a). Therefore, downregulation of the *adc* operon might be due to the polar effect of *adcR* deletion on the downstream genes of *adcR* (Shafeeq et al., 2011a). We further validated our DNA microarray data by qRT-PCR. qRT-PCR data is also in agreement with our transcriptome data (Supplementary data: Table S2).

**Table 4:** **Summary of transcriptome comparison of ***S. pneumoniae*** D39 wild-type with Δ***adcR*** (SS200) grown in CDM with 0.3 mM Ni^2+^**.

**Gene tag^a^**	**Function^b^**	**Ratio^c^**	***P*-value**
*SPD0126*	Pneumococcal surface protein A, PhpA	2.29	1.35E−05
*SPD0277*	6- phospho-beta-glucosidase, CelA	12.36	2.28E−13
*SPD0278*	Hypothetical protein	6.67	1.12E−09
*SPD0279*	PTS system, IIB component, CelB	7.82	3.99E−09
*SPD0280*	Transcriptional regulator, CelR	10.24	2.71E−12
*SPD0281*	PTS system, IIA component, CelC	4.80	1.75E−07
*SPD0282*	Hypothetical protein	6.8	6.87E−10
*SPD0283*	PTS system, IIC component, CelD	7.10	8.67E−09
*SPD0308*	ATP-dependent Clp protease, ATP-binding subunit, ClpL	4.21	5.54E−10
*SPD0888*	Adhesion lipoprotein, AdcAII (LmB)	1.65	3.39E−04
*SPD0889*	Pneumococcal histidine triad protein D, PhtD	3.51	1.21E−08
*SPD0893*	Hypothetical protein	3.51	8.62E−07
*SPD1038*	Pneumococcal histidine triad protein A, PhtA	5.59	8.67E−09
*SPD1514*	ABC transporter, ATP-binding protein	−3.35	4.04E−08
*SPD1515*	Hypothetical protein	−4.06	4.50E−09
*SPD1516*	Hypothetical protein	−4.57	3.25E−09
*SPD1997*	Zinc ABC transporter, zinc-binding lipoprotein, AdcA	−18.45	4.07E−13
*SPD1998*	Zinc ABC transporter, permease protein, AdcB	−2.71	1.29E−04
*SPD1999*	Zinc ABC transporter, ATP-binding protein, AdcC	−10.76	3.21E−12
*SPD2000*	*adc* operon repressor, AdcR	−15.29	7.99E−11
*SPD2001*	Hypothetical protein	−25.05	1.31E−12

a *Gene numbers refer to D39 locus tags*.

b *D39 annotation/TIGR4 annotation (Hoskins et al., 2001; Lanie et al., 2007)*.

c *Ratios >2.0 or < 2.0 (SS200+0.3 mM Ni^2+^/wild-type + 0.3 mM Ni^2+^)*.

### Binding of AdcR to its target is Zn^2+^-and Ni^2+^-dependent

**Figure 5:**
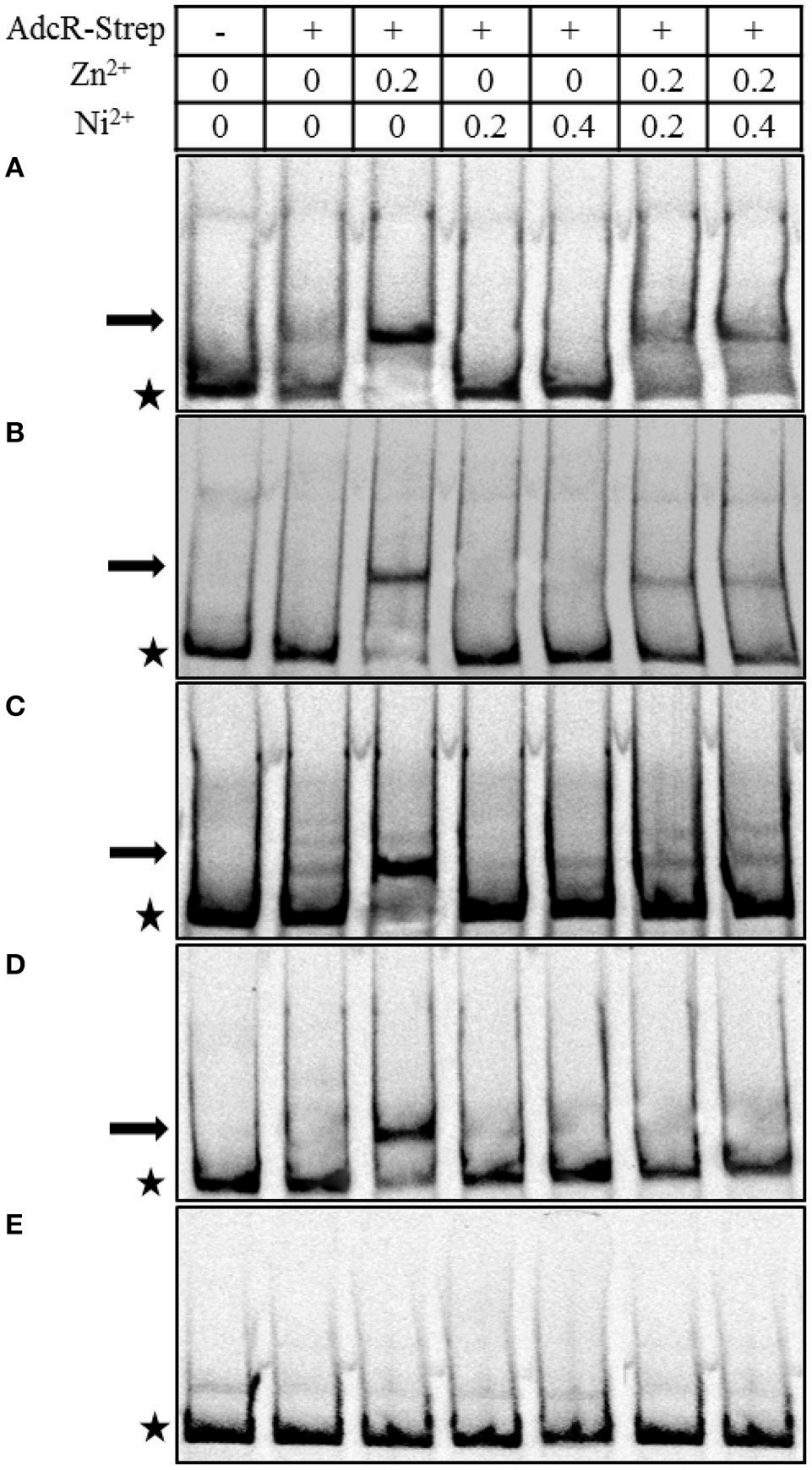
*****In vitro*** interaction of Ad-Strep tag with the promoter regions of ***adcR*** (A), ***adcAII*** (B), ***phtA*** (C), ***phtB*** (D), and ***pcpA*** (E)**. Ad-Strep was added at a concentration of 30 nM as indicated above panel, while lane 1 is without added protein. Arrows indicate the position of shifted probe and asterisks indicate the position of free probe. 0.2 mM Zn^2+^ was added in lanes 3, 6, and 7. Whereas, Ni^2+^ was added at the concentration of 0.2 mM in lane 4 and 6, and 0.4 mM in lanes 5 and 7.

To study the direct interaction of AdcR with the promoter regions of the genes belonging to the AdcR regulon in the presence of Ni^2+^, we performed EMSAs with purified Strep-tagged AdcR (Ad-Strep tag) and ^33^P-labeled promoters of *adcR, adcAII, phtA, phtB*, and *pcpA*. To prevent the interference of metal ions with Ad-Strep tag, all the experiments were performed in EDTA free gels and buffers. The *pcpA* promoter region was taken as a negative control. Ad-Strep tag was unable to shift the promoter regions of *adcR, adcAII, phtA*, and *phtB* in the absence of metal ions (Lane 2 in Figure 5). However, the addition of 0.2 mM Zn^2+^ led to the binding of Ad-Strep tag to the promoter regions of *adcR, adcAII, phtA*, and *phtB* (Lane 3 in Figures 5A–D), which is consistent with our previous study (Shafeeq et al., 2011a). Interestingly, 0.2 and 0.4 mM Ni^2+^ were unable to stimulate the binding of Ad-Strep tag with the promoter regions of *adcR, adcAII, phtA*, and *phtB* (Lane 4 and 5 in Figures 5A–D). In our transcriptome data mentioned above, Ni^2+^ showed a derepressive effect on the expression of the AdcR regulon. Therefore, we also decided to check the interaction of Ad-Strep tag with the promoter regions of *adcR, adcAII, phtA*, and *phtB* in the presence of both Zn^2+^ and Ni^2+^ together. The Zn^2+^-dependent interaction of AdcR with these promoters in the presence of 0.2 mM Zn^2+^ was alleviated with the addition of 0.2 mM or 0.4 mM Ni^2+^ (Lane 6 and 7 in Figures 5A–D). Under the same conditions, we did not see any band shift with the promoter region of *pcpA* as a negative control (Figure 5E). Thus, this data indicates that Zn^2+^ and Ni^2+^ have an opposite effects on the interaction of AdcR with the promoter regions of *adcR, adcAII, phtA*, and *phtB*.

### Effect of Ni^2+^on SczA-mediated expression of the Zn^2+^-efflux system *czcD*

**Figure 6:**
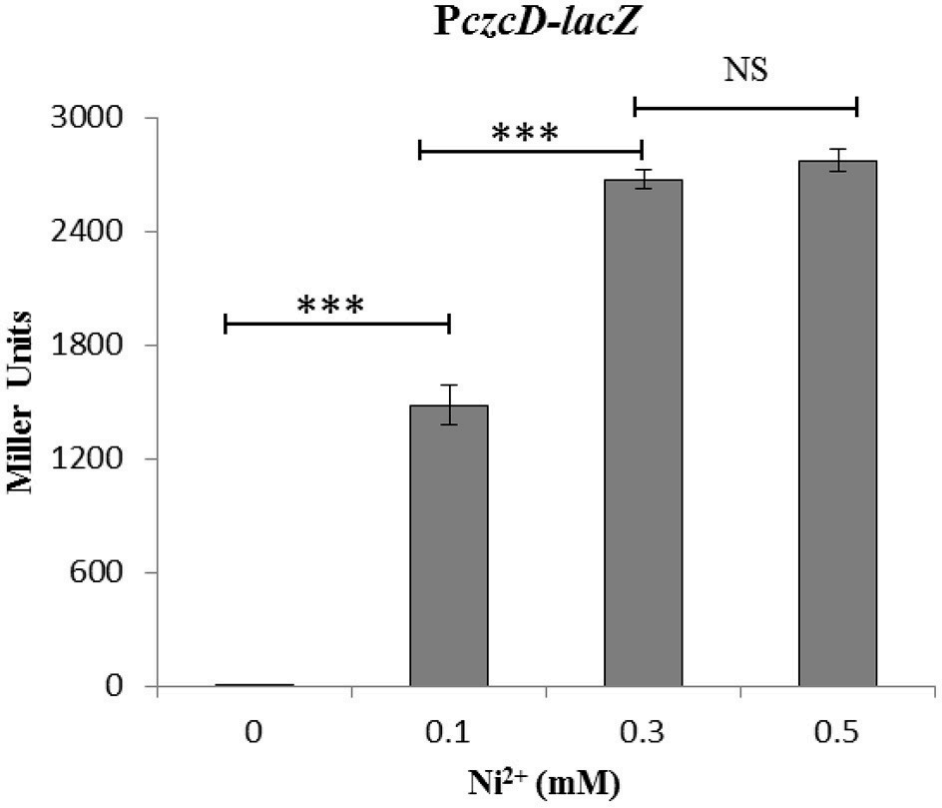
**Expression level (miller units) of the D39 wild-type containing transcriptional ***lacZ***-fusion to P***czcD*** grown in CDM with different added concentrations of Ni^2+^**. Standard deviation of three independent replications is indicated with error bars. Statistical significance of the differences in the expression levels was determined by One-way ANOVA (NS, not significant and ^***^P < 0.0001).

To investigate the regulation of *czcD* in the presence of Ni^2+^, we studied the transcriptional response of P*czcD-lacZ* grown in complete CDM with the addition of different concentrations of Ni^2+^. β-galactosidase assays showed that P*czcD-lacZ* responded to Ni^2+^ and its expression was highly increased with an increasing concentration of Ni^2+^ (Figure 6). This data is in agreement with our transcriptomic data mentioned above and suggests the putative role of CzcD in Ni^2+^ homeostasis.

## Discussion

Transition metal ions such as Mn^2+^, Zn^2+^, Cu^2+^, Fe^2+^, Co^2+^, and Cd^2+^ have been shown to play a pivotal role in the metabolism and virulence of *S. pneumoniae* (Brown et al., 2001; Kloosterman et al., 2008; Shafeeq et al., 2011b; Begg et al., 2015). However, the role of Ni^2+^ on the global gene expression of *S. pneumoniae* has not been studied before. In this study, we analyze the transcriptome changes in *S. pneumoniae* D39 wild-type in response to high Ni^2+^ concentration. The expression of a number of important genes and operons with diverse functions, including the AdcR regulon (*adcRCBA, adcAII*-*phtD, phtA, phtB*, and *phtE*), the PsaR regulon (*pcpA, prtA*, and *psaBCA*) regulon, and the Zn^2+^-efflux system *czcD* were significantly altered in the presence of Ni^2+^. We further studied the role of Ni^2+^ in the regulation of the AdcR regulon and demonstrated that Ni^2+^ plays an opposite role compared to Zn^2+^ in the regulation of the AdcR regulon.

The AdcR regulon consists of *adcRCBA, adcAII*-*phtD, phtA, phtB, phtE*, and *adhC* in *S. pneumoniae*. The *adc* operon (*adcRCBA*) is involved in Zn^2+^ acquisition, and encodes for a Zn^2+^-responsive MarR family transcriptional regulator, AdcR, two ABC transporter proteins AdcC and AdcB, and an extracellular Zn^2+^-binding protein AdcA (Dintilhac et al., 1997; Dintilhac and Claverys, 1997; Bayle et al., 2011). The *adcAII* gene encodes an adhesion lipoprotein which has an overlapping specificity with AdcA for Zn^2+^ (Bayle et al., 2011). AdcAII belongs to the LraI-lipoprotein family and is organized in an operon with a *phtD* gene encoding pneumococcal histidine triade protein precursor D (PhtD). *phtA, phtB*, and *phtE* encodes for pneumococcal histidine triade protein A, B, and E, respectively. Recent studies have demonstrated the role of the PhT family proteins (PhtA, PhtB, PhtE, and PhtD) in intracellular Zn^2+^ acquisition and pathogenesis in *S. pneumoniae* (Hava and Camilli, 2002; Ogunniyi et al., 2009; Plumptre et al., 2014b). The *adhC* gene encodes for a Zn^2+^-containing alcohol dehydrogenase. Previously, it was demonstrated that the expression of *adcRCBA, adcAII*-*phtD, phtA, phtB*, and *phtE* is repressed, while the expression of *adhC* is activated by the transcriptional regulator AdcR in the presence of Zn^2+^ (Shafeeq et al., 2011a). Here, we show that Ni^2+^ also plays a role in the regulation of *adcRCBA, adcAII*-*phtD, phtA, phtB*, and *phtE*. Our β-galactosidase assays showed that the expression of *adcRCBA, adcAII*-*phtD, phtA, phtB*, and *phtE* was increased with increasing concentrations of Ni^2+^. However, we did not find any significant change in the expression of *adhC* in our both transcriptome analysis performed in this study. This might exclude the role of Ni^2+^ in the AdcR mediated regulation of *adhC*.

High concentrations of Ni^2+^ can be very toxic for bacteria (Macomber and Hausinger, 2011). Therefore, bacteria must limit the toxic amount of Ni^2+^ to perform normal cellular functions. In many bacteria, CDF-family efflux pumps help to maintain proper concentrations of heavy metals in the cell. For example, in *Bacillus subtilis*, the CzcD heavy metal efflux pump is involved in the homeostasis of Zn^2+^, Co^2+^, Cu^2+^, and Ni^2+^, and is regulated by CzrA (Moore et al., 2005). It is also important to note that the expression of *czcD* is highly upregulated in our transcriptome analysis in response to Ni^2+^. Expression of *czcD* is regulated by the TetR family transcriptional regulator SczA in the presence of Zn^2+^, Co^2+^, or Ni^2+^ (Kloosterman et al., 2007). Moreover, Zn^2+^, Co^2+^, or Ni^2+^ has been shown to stimulate the binding of SczA to the promoter region of *czcD* (Kloosterman et al., 2007). In this study, we further confirmed the expression of *czcD* in the presence of Ni^2+^ by transcriptional *lacZ*-reporter study with P*czcD*-*lacZ* and our results are consistent with a previous study (Kloosterman et al., 2007).

The PsaR regulon consists of *psaBCA, pcpA*, and *prtA* that encodes for the Mn^2+^ uptake system (PsaBCA), a choline binding protein (PcpA), and a serine protease (PrtA), respectively. The expression of the PsaR regulon is shown to be repressed by the DtxR family transcriptional regulator PsaR in the presence of Mn^2+^ (Johnston et al., 2006). Notably, Zn^2+^ and Co^2+^ can bind with PsaR to relieve the Mn^2+^-dependent repression of the PsaR regulon (Kloosterman et al., 2008; Manzoor et al., 2015a). Recently, we have studied the regulation of the PsaR regulon in the presence of Ni^2+^ and demonstrated that like Zn^2+^ and Co^2+^, Ni^2+^ also has the ability to derepress the Mn^2+^-dependent repression of the PsaR regulon, and that high concentrations of Ni^2+^ leads to cell-associated Mn^2+^ deficiency (Manzoor et al., 2015b). In this study, we have also observed the significant upregulation of the PsaR regulon in our transcriptome analysis performed in the presence of Ni^2+^ (Table 3). Upregulation of the PsaR regulon in our transcriptome further verifies our previous results (Manzoor et al., 2015b). Moreover, we have also observed the cell-associated deficiency of Mn^2+^ in our ICP-MS analysis performed in this study (Figure 1), which is also in consistent with our previous results (Manzoor et al., 2015b).

The interplay, or competition, of metal ions plays an important role in the regulation of metal responsive genes. In *S. pneumoniae*, competition of Mn^2+^ with Zn^2+^, Co^2+^, or Ni^2+^ in the regulation of the PsaR regulon by transcriptional regulator PsaR has already extensively been studied (Kloosterman et al., 2008; Manzoor et al., 2015a,b). Similarly, the interplay of Cu^2+^ and Zn^2+^ in the regulation of *cop* operon by transcriptional regulator CopY was studied before, where Cu^2+^ induces and Zn^2+^ represses the CopY-mediated expression of *cop* operon (Shafeeq et al., 2011b). Here, we elaborated for the first time the interplay of Ni^2+^ and Zn^2+^ in the regulation of genes belonging to the AdcR regulon. Our *lacZ*-reporter studies determined the ability of Ni^2+^, in derepressing the Zn^2+^-dependent repression of *adcRCBA, adcAII*-*phtD, phtA, phtB*, and *phtE*. Our *in vitro* data showed that the Zn^2+^-dependent binding of AdcR to the promoter regions of the genes belonging to the AdcR regulon was alleviated by the addition of Ni^2+^. Recently, it has been shown that Cd^2+^-uptake reduces the accumulation of cell-associated Mn^2+^ and Zn^2+^ (Begg et al., 2015). Our ICP-MS comparison of cells grown in CDM with 0.5 mM to 0 mM Ni^2+^ has not shown any difference in the concentration of Zn^2+^ or other metal ions, which also indicates the direct role of Ni^2+^ in the regulation of *adcRCBA, adcAII*-*phtD, phtA, phtB*, and *phtE*. Moreover, the role of genes belonging to the AdcR regulon in the pathogenesis of *S. pneumoniae* has already been demonstrated, which also suggests the important role of Ni^2+^ in pneumococcal virulence.

### Conflict of interest statement

The authors declare that the research was conducted in the absence of any commercial or financial relationships that could be construed as a potential conflict of interest.
