# Supplementary material for: The Regulation of the AdcR Regulon in Streptococcus pneumoniae Depends Both on Zn2+- and Ni2+-Availability
Source: Front Cell Infect Microbiol. 2015 Dec 8;5:91. doi: 10.3389/fcimb.2015.00091 (PMC4672087; doi:10.3389/fcimb.2015.00091)
Supplement: Supplementary file 1 [file DataSheet1.docx]

**Supplementary Data**

**The regulation of the AdcR regulon in *Streptococcus pneumoniae* depends both on Zn^2+^- and Ni^2+^ -availability**

Irfan Manzoor, Sulman Shafeeq, Muhammad Afzal, and Oscar P. Kuipers

**Material and Methods**

**RNA extraction purification for quantitative RT-PCR**

Total RNA was isolated from *S. pneumoniae* D39 wild-type and Δ*adcR* strains grown in CDM with and without the addition of Ni^2+^ as described (Shafeeq et al., 2011). DNA contamination from RNA samples was removed by the treatment of 2U of RNase free Dnase I (Invitrogen, Paisley, United Kingdom). First, strand cDNA synthesis was performed on RNA (Shafeeq et al., 2011; Yesilkaya et al., 2008). cDNA (2 µl) was amplified in a 20 µl reaction volume that contained 3 pmol of each primer (Table S3) and the reactions were performed in triplicate (Shafeeq et al., 2011). The transcription level of specific genes was normalized to *gyrA* transcription, amplified in parallel with gyrA-F and gyrA-R primers. The results were interpreted using the comparative CT method (Schmittgen and Livak, 2008).

**Table S1:** The relative expression of the genes belonging to the AdcR regulon and CzcD. The expression of all genes was normalized with the housekeeping gene *gyrA*. Results represent the mean and standard deviation of three independent experiments. The fold increase is relative to the expression in 0.5 mM Ni^2+^ with 0 mM Ni^2+^ grown in CDM.

| **Gene tag** | **Gene function** | **Fold Raito** |  |
| --- | --- | --- | --- |
| *SPD0888* | Adhesion lipoprotein, AdcAII (LmB) | 8.16 (0.36) | |
| *SPD0889* | Pneumococcal histidine triad protein D precursor, PhtD | 6.41 (0.04) | |
| *SPD0890* | Pneumococcal histidine triad protein E precursor, PhtE | 7.22 (0.08) | |
| *SPD1038* | Pneumococcal histidine triad protein A precursor, PhtA | 12.20 (0.12) | |
| *SPD1635* | Galactose operon repressor, GalR | 52.02 (10) | |
| *SPD1997* | Zinc ABC transporter, zinc-binding lipoprotein, AdcA | 11.12 (0.66) | |
| *SPD1998* | Zinc ABC transporter, permease protein, AdcB | 6.56 (0.14) | |
| *SPD1999* | Zinc ABC transporter, ATP-binding protein, adcC | 13.42 (0.28) | |
| *SPD2000* | *adc* operon repressor, AdcR | 7.12 (0.56) | |

**Table S2:** The relative expression of the genes belonging to the AdcR regulon and CzcD. The expression of all genes was normalized with the housekeeping gene *gyrA*. Results represent the mean and standard deviation of three independent experiments. The fold increase is relative to the expression in 0.5 mM Ni^2+^ with 0 mM Ni^2+^ grown in CDM.

| **Gene tag** | **Gene function** | **Fold Raito** |  |
| --- | --- | --- | --- |
| *SPD0888* | Adhesion lipoprotein, AdcAII (LmB) | 3.66 (0.36) | |
| *SPD0889* | Pneumococcal histidine triad protein D precursor, PhtD | 11.54 (0.08) | |
| *SPD1038* | Pneumococcal histidine triad protein A precursor, PhtA | 6.10 (0.12) | |
| *SPD1997* | Zinc ABC transporter, zinc-binding lipoprotein, AdcA | -45.22 (9) | |
| *SPD1998* | Zinc ABC transporter, permease protein, AdcB | -36.33 (8) | |
| *SPD1999* | Zinc ABC transporter, ATP-binding protein, adcC | -58.50 (10) | |
| *SPD2000* | *adc* operon repressor, AdcR | -60.40 (12) | |

**Table S3:** List of primers used for qRT-PCR analysis.

| Name | Nucleotide Sequence (5’🡪3’) |
| --- | --- |
| czcD_Fr | TCTAGCGCTGTTCTTGCTGA |
| czcD_Rv | AACAGAGCCCGTTACGAGAA |
| adcAII_Fr | GCAGCCATCTATGATGCAGA |
| adcAII_Rv | AAAAGCTGGAGAAGAAGCCC |
| phtE_Fr | CGTTCCTCATGGAGGTCACT |
| phtE_Rv | GGATCAACTAGCAAGCCAGC |
| phtD_Fr | AATGCAAATCCAGCTCAACC |
| phtD_Rv | TGATGGCCTTATTTTCGACC |
| phtA_Fr | GCGGCAGCTTTGATTTTAAG |
| phtA_Rv | TGATGAGGTTAGCAAGCGTG |
| phtB_Fr | GAGGTTAGCAAGCGTGAAGG |
| phtB_Rv | TTGCTGATGAAGGATCCAAA |
| adcR_Fr | GCGGAAAATCAGCATGAAAT |
| adcR_Rv | CAAGGAAGGGATGTTGGAAA |
| adcC_Fr | ATCCGAGAAAAGGCTGGTTC |
| adcC_Rv | TTGCTTCTGACCCTGATGTG |
| adcB_Fr | TGGGAACCTTCCTCATCTTG |
| adcB_Rv | TGGAAATCGGGACAGCTATC |
| adcA_Fr | ATACGAACCATCTGCCAAGG |
| adcA_Rv | GAAGAGGGAGACCATGACCA |
| gyrA-Fr | CGAGGCACGTATGAGCAAGA |
| gyrA-Rv | GACCAAGGGTTCCCGTTCAT |

**References**

Schmittgen, T. D., and Livak, K. J. (2008). Analyzing real-time PCR data by the comparative C(T) method. *Nat.Protoc.* 3, 1101–1108.

Shafeeq, S., Yesilkaya, H., Kloosterman, T. G., Narayanan, G., Wandel, M., Andrew, P. W., et al. (2011). The cop operon is required for copper homeostasis and contributes to virulence in Streptococcus pneumoniae. *Mol. Microbiol.* 81, 1255–1270.

Yesilkaya, H., Manco, S., Kadioglu, A., Terra, V. S., and Andrew, P. W. (2008). The ability to utilize mucin affects the regulation of virulence gene expression in Streptococcus pneumoniae. *FEMS Microbiol. Lett.* 278, 231–235.
